# A nomogram composed of clinicopathologic features and preoperative serum tumor markers to predict lymph node metastasis in early gastric cancer patients

**DOI:** 10.18632/oncotarget.10732

**Published:** 2016-07-20

**Authors:** Lin-Yong Zhao, Yuan Yin, Xue Li, Chen-Jing Zhu, Yi-Gao Wang, Xiao-Long Chen, Wei-Han Zhang, Xin-Zu Chen, Kun Yang, Kai Liu, Bo Zhang, Zhi-Xin Chen, Jia-Ping Chen, Zong-Guang Zhou, Jian-Kun Hu

**Affiliations:** ^1^ Department of Gastrointestinal Surgery, West China Hospital, Sichuan University, China; ^2^ Laboratory of Gastric Cancer, State Key Laboratory of Biotherapy/Collaborative Innovation Center of Biotherapy, West China Hospital, Sichuan University, China; ^3^ West China School of Medicine, Sichuan University, China

**Keywords:** early gastric cancer, nomogram, lymph node metastasis, prediction, tumor markers

## Abstract

Predicting lymph node metastasis (LNM) accurately is of great importance to formulate optimal treatment strategies preoperatively for patients with early gastric cancer (EGC). This study aimed to explore risk factors that predict the presence of LNM in EGC. A total of 697 patients underwent gastrectomy enrolled in this study, were divided into training and validation set, and the relationship between LNM and other clinicopathologic features, preoperative serum combined tumor markers (CEA, CA19-9, CA125) were evaluated. Risk factors for LNM were identified using logistic regression analysis, and a nomogram was created by R program to predict the possibility of LNM in training set, while receiver operating characteristic (ROC) analysis was applied to assess the predictive value of the nomogram model in validation set. Consequently, LNM was significantly associated with tumor size, macroscopic type, differentiation type, ulcerative findings, lymphovascular invasion, depth of invasion and combined tumor marker. In multivariate logistic regression analysis, factors including of tumor size, differentiation type, ulcerative findings, lymphovascular invasion, depth of invasion and combined tumor marker were demonstrated to be independent risk factors for LNM. Moreover, a predictive nomogram with these independent factors for LNM in EGC patients was constructed, and ROC curve demonstrated a good discrimination ability with the AUC of 0.847 (95% CI: 0.789-0.923), which was significantly larger than those produced in previous studies. Therefore, including of these tumor markers which could be convenient and feasible to obtain from the serum preoperatively, the nomogram could effectively predict the incidence of LNM for EGC patients.

## INTRODUCTION

The incidence of early gastric cancer (EGC), defined as adenocarcinoma limited to the mucosa or submucosa of the stomach, irrespective of lymph node metastasis (LNM), has been increasing worldwide.[1–3] Apart from gastrectomy with lymphadenectomy, endoscopic surgical techniques including of endoscopic mucosal resection (EMR) and endoscopic submucosal dissection (ESD) have gained increasing popularity and have been widely regarded as an alternate treatment for some EGC patients [4, 5], from which patients can avoid a potentially morbid surgical procedure and preserve stomach function as well as maintain high postoperative quality of life.[6–9] Nevertheless, endoscopic resection with curative intent should only be considered with the absence of regional lymph node metastases, as regional lymph nodes are untreated in this procedure.[10, 11] Thus, identifying the risk factors for LNM is of crucial importance to determine the optimal treatment for EGC patients.

Previous studies suggested that some clinicopathologic features, such as differentiated type, depth of invasion, tumor size and the presence of ulceration [12–15], and biological markers including of P53, ki67, Her-2 and E-cad [16, 17], were the independent risk factors for LNM, even if unanimous agreement has not been reached. However, there were few studies evaluating the correlation between the preoperative serum tumor markers (CEA, CA125, CA19-9) and LNM in EGC [18, 19], and nomogram has been applied to quantify risk factors of LNM in several carcinomas other than EGC [20, 21]. Furthermore, there is no predictive nomogram analyzing the clinicopathologic features and preoperative serum tumor markers for the risk of LNM in EGC. Therefore, the aim of this study was to identify risk factors for LNM and to construct a nomogram based on these factors for EGC patients to guide treatment.

## RESULT

### Correlation analysis between the clinicopathologic features and lymph node metastasis (LNM)

There were a total of 697 early gastric cancer (EGC) patients enrolled in this study, including 446 male patients and 251 female patients. The average age was 56.6 years old (range, 25-83 years old). 598 patients were enrolled in the training set, with 447 patients in LNM (−) group and 151 patients in LNM (+) group, while 99 patients were divided into the validation set, with 67 patients in LNM (−) group and 32 patients in LNM (+) group. Difference in terms of all the clinicopathologic features, was not found to be significant between the training set and validation set (all the p* value >0.05), indicating a similar constitution and a balanced baseline between them.

As shown in Table 1, LNM was found to be significantly associated with tumor size, macroscopic type, differentiation type, ulcerative findings, lymphovascular invasion, depth of invasion and combined tumor marker both in the training set and validation set. To be specific, there were significantly more patients with larger tumor size, depressed/mixed macroscopic type, undifferentiated type, submucosa invasion, the presence of ulcerative findings or combined tumor marker in LNM (+) group than those in LNM (−) group.

**Table 1:** Correlation between lymph node metastasis and clinicopathologic features. n(%)

Factors	Training set				Validation set				P^*^
	LNM (−) (n=447)	LNM (+) (n=151)	Total (n=598)	P	LNM (−) (n=67)	LNM (+) (n=32)	Total (n=99)	P	
Gender				0.075				0.858	0.251
Male	302(67.6)	90(59.6)	392(65.6)		39(58.2)	20(62.5)	59(59.6)		
Female	145(32.4)	61(40.4)	206(34.4)		28(41.8)	12(37.5)	40(40.4)		
Age				0.080				0.262	0.070
<60	263(58.8)	101(66.9)	364(60.9)		45(67.2)	25(78.1)	70(70.7)		
≥60	184(41.2)	50(33.1)	234(39.1)		22(32.8)	7(21.9)	29(29.3)		
Tumor location				0.142				0.326	0.059
Upper third	55(12.3)	10(6.6)	65(10.9)		6(9.0)	2(6.3)	8(8.1)		
Middle third	78(17.4)	26(17.2)	104(17.4)		21(31.3)	6(18.8)	27(27.3)		
Lower third	314(70.2)	115(76.2)	429(71.7)		40(59.7)	24(74.9)	64(64.6)		
Tumor size				<0.001				0.047	0.068
≥2cm	319(71.4)	137(90.7)	456(76.3)		41(61.2)	26(81.3)	67(67.7)		
<2cm	128(28.6)	14(9.3)	142(23.7)		26(38.8)	6(18.7)	32(32.3)		
Count of lymph node				0.186				0.087	0.871
≥15	306(68.5)	112(74.2)	418(69.9)		51(76.1)	19(59.4)	70(70.7)		
<15	141(31.5)	39(25.8)	180(30.1)		16(23.9)	13(40.6)	29(29.3)		
Macroscopic type				0.014				0.027	0.082
Elevated/Flat	293(65.5)	82(54.3)	375(62.7)		41(61.2)	12(37.5)	53(53.5)		
Depressed/Mixed	154(34.5)	69(45.7)	223(37.3)		26(38.8)	20(62.5)	46(46.5)		
Differentiation type				0.010				0.026	0.072
Differentiated	320(71.6)	91(60.3)	411(68.7)		45(67.2)	14(38.1)	59(59.6)		
Undifferentiated	127(28.4)	60(39.7)	187(31.3)		22(32.8)	18(61.9)	40(40.4)		
Ulcerative findings				0.002				0.004	0.216
Absent	322(72.0)	88(58.3)	410(68.6)		56(83.6)	18(38.1)	74(74.7)		
Present	125(28.0)	63(41.7)	188(31.4)		11(16.4)	14(61.9)	25(25.3)		
Lymphovascular invasion				0.012				0.021	0.314
Absent	373(89.4)	112(74.2)	485(81.1)		56(83.6)	20(62.5)	76(76.8)		
Present	74(10.6)	39(25.8)	113(18.9)		11(16.4)	12(37.5)	23(23.2)		
Depth of invasion				<0.001				0.020	0.058
Mucosa(T1a)	247(55.3)	44(29.1)	291(48.7)		31(46.3)	7(21.9)	38(38.4)		
Submucosa(T1b)	200(44.7)	107(70.9)	307(51.3)		36(53.7)	25(78.1)	61(61.6)		
Combined tumor marker				0.004				0.021	0.223
Positive	69(18.3)	39(25.8)	108(18.1)		11(16.4)	12(37.5)	23(23.2)		
Negative	378(81.7)	112(74.2)	490(81.9)		56(83.6)	20(62.5)	76(76.8)		

LNM(+)/(−): the presence/absence of lymph node metastasis;

P* the difference between the training set and the validation.

### Identification of risk factors and multivariate analysis for LNM

As illustrated in Table 2, logistic regression analysis was performed to determine the risk factors for LNM. In the univariate analysis, the involved factors were significantly composed of clinicopathologic features, such as tumor size (OR=2.392, p<0.001), macroscopic type (OR=1.326, p=0.021), differentiation type (OR=3.432, p=0.011), ulcerative findings (OR=2.124, p=0.007), lymphovascular invasion (OR=2.380, p=0.006), depth of invasion (OR=2.931, p<0.001), combined tumor markers (OR=1.975, p=0.001). Additionally, multivariate analysis illustrated that tumor size (OR=1.254, p=0.011), differentiation type (OR=2.832, p=0.027), ulcerative findings (OR=1.656, p=0.005), lymphovascular invasion (OR=1.775, p=0.023), depth of invasion (OR=2.320, p<0.001) and combined tumor marker (OR=1.231, p=0.034) were independent risk factors for LNM. And there were no significant differences between age, gender, tumor location, count of lymph node and LNM.

**Table 2:** Logistic regression analysis of the risk factors for lymph node metastasis

Factors	Univariate analysis		Multivariate analysis	
	OR(95%CI)	P value	OR(95%CI)	P value
Gender	1.280(0.850-1.928)	0.237	-	-
Age	1.239(0.813-1.888)	0.319	-	-
Tumor location	1.465(0.967-2.219)	0.062	-	-
Tumor size	2.392(1.765-4.234)	<0.001	1.254(1.011-1.981)	0.011
Count of lymph node	1.171(0.989-1.828)	0.073	-	-
Macroscopic type	1.326(1.183-1.818)	0.021	1.412(0.853-1.729)	0.181
Differentiation	3.432(2.900-4.963)	0.011	2.832(2.090-3.709)	0.027
Ulcerative findings	2.124(1.975-2.721)	0.007	1.656(1.007-2.092)	0.005
Lymphovascular invasion	2.380(1.569-2.763)	0.006	1.775(1.103-2.121)	0.023
Depth of invasion	2.931(1.634-3.921)	<0.001	2.320(1.923-3.112)	<0.001
Combined tumor markers	1.975(1.665-2.240)	0.001	1.231(1.015-1.675)	0.034

OR: odds ratio; CI: confidence interval

### The nomogram for predicting the LNM

**Figure 1:**
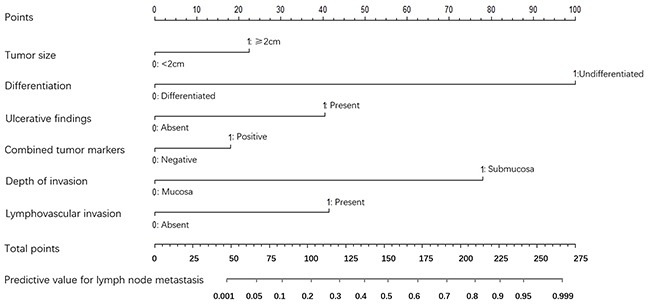
A nomogram composed of all the independent risk factors to predict the probability of lymph node metastasis for patients with early gastric cancer The risk value of lymph node metastasis was calculated by drawing a vertical line to the point on the axis for each of the factors. The points for each factor were summed and located on the total point line. And then, the bottom line corresponding vertically to the above total line illustrated the individual predictive value for lymph node metastasis.

Nomogram was furtherly constructed by these independent risk factors in the training set to predict the LNM for patients with EGC. This nomogram model based on these risk factors which could affect the incidence of LNM was displayed in Figure 1. For each patient, points were assigned for each of these clinicopathologic risk factors (tumor size, differentiation type, ulcerative findings, lymphovascular invasion, depth of invasion and combined tumor marker), while a total point, calculated from the nomogram, was visually corresponded to a predictive value for LNM.

**Figure 2:**
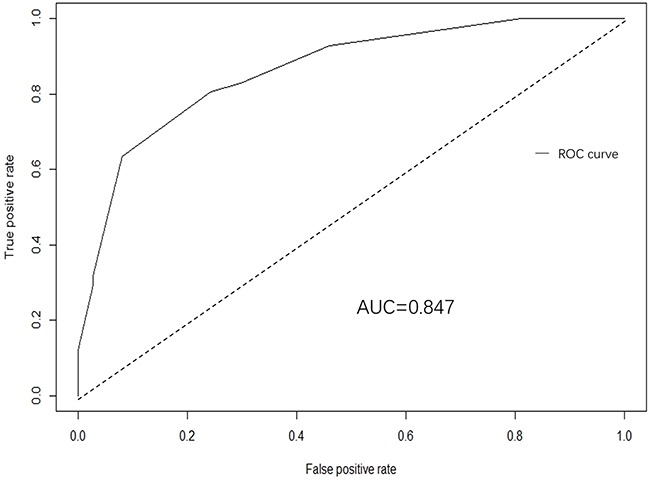
A receiver operating characteristics (ROC) curve of the multivariate logistic regression model illustrated an AUC of 0.847 (95% CI: 0.789-0.923), which revealed a good concordance and a reliable ability to estimate the status of lymph nodal involvement

**Figure 3:**
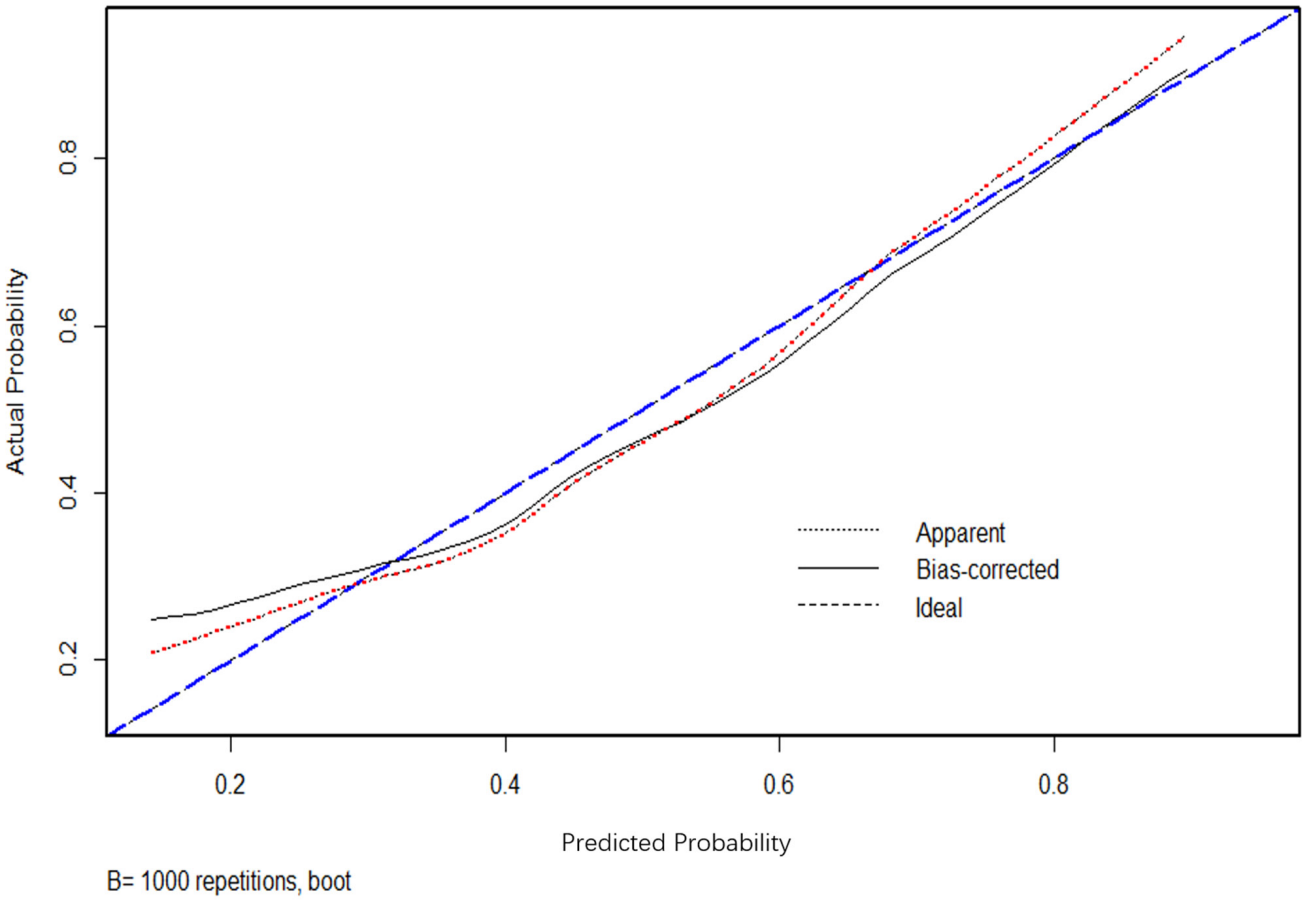
Calibration plot of nomogram Dotted line (blue) indicated the ideal nomogram in which predicted and actual probabilities were perfectly identical; Dashed line (red) indicated actual nomogram performance with apparent accuracy; Solid line (black) presented bootstrap corrected performance of our nomogram, scatter estimate of future accuracy.

In addition, ROC curve and calibration plot were displayed to validate the predictive accuracy of the nomogram model. Specifically, ROC in Figure 2 illustrated an AUC of 0.847 (95% CI: 0.789-0.923), which revealed a good concordance and a reliable ability to estimate the status of lymph nodal involvement. Besides, calibration plot in Figure 3 showed the performance characteristics of the nomogram. The x-axis was the prediction calculated with the nomogram while the y-axis was the actual prediction for LNM. In the plot, dotted line (blue) indicated the ideal nomogram in which predicted and actual probabilities were perfectly identical, whereas dashed line (red) indicated actual nomogram performance with apparent accuracy and solid line (black) presented bootstrap corrected performance of our nomogram, scatter estimate of future accuracy. Note that the predicted probability calculated using the nomogram corresponded accurately to the actual outcomes, because that the solid line was close to the dotted line.

In order to assess whether this model was indeed trustworthy and evaluate how much improvement was gained using these clinicopathologic features and biomarkers in this study, we also validated several predictive models, composed of different factors which were reported in previous studies [7, 22–25], to generate several corresponding areas under the curve (AUC), as shown in Table 3. Furtherly, we compared them to the AUC produced by our predictive model in our study, and found that the AUC value was significantly larger than those produced in previous studies (all p<0.05, Table 3).

**Table 3:** Comparison and validation of different models for LNM

Authors (ref.)	Including factors	AUC(95%CI)	P
Zheng ZX et al [22]	T, Ts, Diff., Ulcer, LVI, Age, Macroscopic type	0.811(0.763-0.877)	<0.05
Ahmad et al [23]	LVI, T	0.684(0.648-0.746)	<0.05
Lee H. et al [7]	Tumor location, Ulcer	0.649(0.603-0.695)	<0.05
Li Hua et al [25]	LVI, Diff., T, Ts	0.795(0.723-0.858)	<0.05
Holscher et al [24]	Ts, Diff., T	0.738(0.673-0.785)	<0.05

T: depth of invasion; Ts: tumor size; Diff.: tumor differentiation; Ulcer: ulcerative findings;

LVI: lymphovascular invasion; AUC: area under the curve; CI: confidential interval.

## DISCUSSION

Predicting lymph node metastasis (LNM) accurately is of great importance to formulate optimal treatment strategies preoperatively for patients with early gastric cancer (EGC). This study evaluating a number of EGC patients revealed detailed data on LNM risk factors and developed a nomogram to predict the risk value for LNM in EGC patients.

In this study, various factors, such as tumor size, differentiation type, ulcerative findings, lymphovascular invasion, depth of invasion were independent risk factors for LNM. To be specific, large tumor size was reported to be generally characterized by aggressive tumor behaviors, which were significantly related to disadvantages in overall survival.[26] The depth of tumor invasion, which could reflect the progression of a primary tumor originating from the mucosal layer, was significantly correlated to the presence of LNM in EGC.[13] In this present study, patients in LNM group were found to be more frequently with larger tumor size (≥2 cm), deeper tumor invasion (submucosa), which were consistent with previous studies. A majority of studies showed that patients with poorly differentiation type and ulcerative findings had higher rates of LNM,[22, 27–30] having a poor prognosis, while some authors insisted that differentiation type and ulcerative findings were not significant associated with LNM, [15, 16, 31] being not prognostic factors for EGC patients. Our findings suggested that LNM were more likely to appear in patients with undifferentiated type, lymphovascular invasion, ulcerative lesions, which were consistent with the former reports. We believed that LNM, as an unfavorable factor, could be correlated with undifferentiated type, and ulcerative lesion in gastric cancer, because of worse biological behavior and tumor progression.

Tumor markers, which could be easily obtained from serum before gastrectomy or endoscopic intervention, were also evaluated in this study. In a recent study, the elevated preoperative serum levels of CEA and CA-153 were illustrated to be independent predictive factors of axillary lymph node metastasis in patients with breast cancer.[32] A previous study revealed that, the tumor makers CA724, CA242, CA199 and CEA were significantly associated with LNM in the gastric patients, and combination of these four tumor markers could be a diagnostic index of LNM. [18] Despite that none of these preoperative tumor markers (CEA, CA19-9 and CA125), which were defined as positive and negative subgroup respectively by cutoff points produced in this study, was individual risk factor for LNM, combined tumor marker proposed in our study which was integrated with these three markers, was demonstrated to be independent risk factor for LNM. Although it did not weigh too much in the nomogram model (OR=1.231, p=0.034), combination work could be more effectively than any of the biomarkers considered alone. Thus, predicting preoperatively the status of lymph nodal involvement could become more feasible than ever before, which is due to the consideration that, as a promising and noninvasive method, monitoring with combination of these serum tumor markers is much more convenient than other factors (e.g. tumor size, differentiation type, depth of invasion, etc.).

Nomogram, corresponding to a predictive model including the independent risk factors that may affect the incidence of LNM, was constructed in our study in the training set. A ROC curve and calibration plot were furtherly developed to validate this nomogram, illustrating a good predictive accuracy, which revealed a good concordance and a reliable ability to estimate the status of lymph nodal involvement. This nomogram provided a helpful method to predict the likelihood of lymph node metastasis for EGC patients, by which individual patient could receive appropriate treatment, e.g. an undifferentiated submucosal EGC patient with the presence of ulcerative findings, tumor size ≥2cm and positive combined tumor marker may have a probability of more than 90% to be together with LNM. So, in this case, gastrectomy with lymphadenectomy but not endoscopic therapy is suggested through this nomogram model. On the contrary, patient with the opposite characteristics should receive endoscopic resection, as the risky value of LNM is lower than 10%. Moreover, in order to evaluate the predictive improvement using these clinicopathologic features and biomarkers in this study, we also validated several previous predictive models and compared them to our model with AUC value, revealing that the AUC value was significantly larger than those produced in previous studies (all p<0.05, Table 3), which illustrated the current model could produce the best prognostic discriminatory ability and predictive accuracy. Therefore, we believe this nomogram model will assist surgeons in formulating the optimal treatment strategy for EGC patients in terms of the probability of LNM.

There were also limitations in our study. Firstly, as a retrospective single-center study, our findings could have been observed by chance, and the optimal cutoff points of serum tumor markers could only make difference in our study. Besides, CA72-4 and CA15-3 were not routinely tested for GC patients in our center before 2012, so they were not evaluated in this study. Furthermore, sample size was not large enough, and external validation with different population should be needed before stronger statement can be done. Moreover, most of the factors enrolled in the nomogram were postoperative variables, only tumor markers could be obtained before surgery, which could limit its use for surgeons to choose the optimal treatment before surgery. However, given that tumor size, differentiation type and ulcerative findings as well as the invasion depth could be roughly measured by preoperative gastroscopy, EUS and CT, we suggested that the endoscopic resection was recommended firstly if the patient was evaluated to be with a low possibility to LNM according to these preoperative findings. After endoscopic resection, additional surgical intervention could be determined using the proposed nomogram model on the basis of a comprehensive review of the endoscopic specimen. Therefore, a surgical strategy should be considered for each patient on a case-by-case basis before the establishment of an accurate preoperative diagnostic method for LNM in early gastric cancer patients.

As shown in our results, the nomogram proposed in this study could effectively predict the incidence of lymph node metastasis for EGC patients, through which surgeons could make optimal treatment strategy for EGC patients.

## PATIENTS AND METHODS

### Patients

The West China Hospital Research Ethics Committee approved the retrospective analysis of anonymous data involved in this study. The data retrieval of this study was based on the Surgical Gastric Cancer Patient Registry in West China Hospital [33]. Patient records were anonymized and de-identified prior to analysis and signed patient informed consent was waived per the committee approval because of the retrospective nature of the analysis.

A total of 697 consecutive EGC patients who received gastrectomy in West China Hospital from January 2000 to December 2015, were retrospectively enrolled in this study. Patients were included on the conditions that: 1) they were histologically proven to be with primary gastric cancer before surgery; 2) Pathological examination confirmed that they had received R0 resection [34], a curative resection with negative residual margins; 3) there were no preoperative distant metastases; 4) The clinicopathologic features and the serum tumor markers including of CEA, CA19-9 and CA125 were clearly recorded. And patients were excluded if they had any of the following situations: 1) with an earlier history of gastrectomy; 2) with any pre-operative chemotherapy or radiotherapy; 3) with positive residual margins; 4) with another malignancy or any other life-threatening diseases diagnosed during three years prior to the operation; 5) death due to postoperative complications in hospital. Finally, of these patients, 598 enrolled from the year 2000 to 2013 were used as the training set, while 99 patients from 2014 to 2015 were regarded as the validation set.

### Definition of combined tumor marker and clinicopathologic features

**Figure 4:**
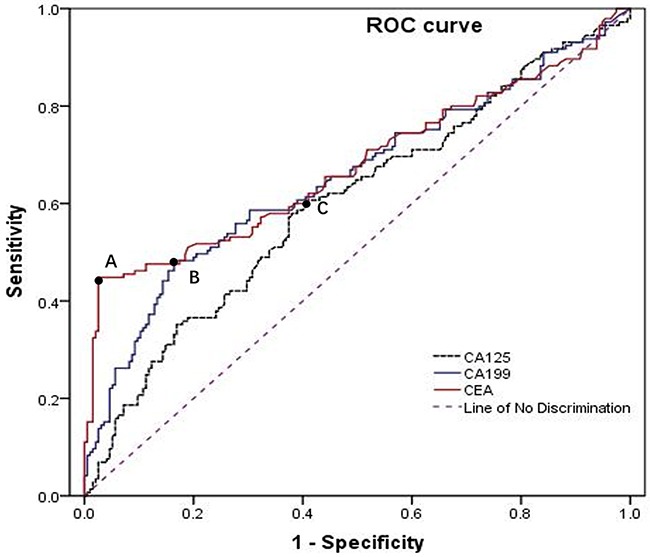
Receiver operating characteristic (ROC) curves showed the optimal cutoff points for CEA, CA19-9 and CA125 were 3.54 ng/ml, 12.83 U/ml, 17.9 6U/ml, corresponding to the *A, B, C black spot,* respectively

Preoperative serum tumor markers, CEA, CA19-9 and CA125, were divided into negative and positive groups respectively by the cutoff points, 3.54ng/ml, 12.83U/ml, 17.96U/ml, produced by ROC analyses (Figure 4). We proposed a new clinicopathologic factor, combined tumor marker, which was composed of the three tumor markers, and it was regarded as positive on the condition that two or three of the tumor markers were found to be positive, while it was defined as negative if two or three of these tumor markers were negative. The clinicopathologic features including of gender, age, tumor location (upper third, middle third, lower third), tumor size (the maximum diameter of the gastric tumor), count of lymph node (number of lymph node retrieved from the surgery), macroscopic type (elevated, flat, depressed, mixed), tumor differentiation (differentiated: well or moderately differentiated adenocarcinomas, undifferentiated: poorly or undifferentiated adenocarcinomas), ulcerative findings, lymphovascular invasion, depth of invasion (mucosa, submucosa), and the combined tumor marker were analyzed in this study. The presence of lymph node metastasis (LNM) was defined as LNM (+), while the absence of LNM was considered as LNM (−).

### Statistical analysis and nomogram construction

All statistical analyses and graphics in this study were demonstrated by the SPSS version 19.0 and R (version3.1.2 *URL* *http://www.R-project.org*/). The optimal cutoff points for CEA, CA19-9 and CA125 were produced using receiver operating characteristic (ROC) analyses. Chi-square test was performed to analyze unordered categorical variables, whereas Mann-Whitney U test was used to evaluate ranked variables. Logistic regression analysis was used to analyze risk factors for LNM, while a nomogram was displayed as a model for predicting the risk of LNM, and it illustrated graphically the factors which could be applied to calculate the risk value of LNM for patients. The predictive accuracy of the nomogram was then validated using ROC and quantified by the area under the curve (AUC). An AUC of 0.5 indicates no relationship while an AUC of 1.0 tells a perfect concordance. [35] Moreover, the nomogram was subjected to 1000 boot strap resamples for reduction of overfit bias and for internal validation with logistic calibration plot. The two-sided p value of less than 0.05 was considered to be statistically significant.
